# Extended criteria deceased donor kidney transplantation in a patient with limited life expectancy

**DOI:** 10.3389/ti.2026.16572

**Published:** 2026-05-01

**Authors:** Aisling E. Courtney, Hannah Magowan, Tim J. Brown

**Affiliations:** Regional Nephrology and Transplant Unit, Belfast City Hospital, Belfast, United Kingdom

**Keywords:** deceased donation, ethics in transplantation, extended criteria, kidney transplantation, palliation

Dear Editors,

The transformational effects of transplantation are universally recognised. For those with kidney failure the benefits compared to dialysis are notable and extend across all socioeconomic classes, all ethnicities, and all age groups.

However the gap between those waiting for a kidney transplant and the relatively static deceased donor pool is increasing. In the UK in 2024–2025 there were almost three times as many people on the waiting list than were transplanted [1].

To maximise utilisation of all donated organs, the UK ‘Fast Track’ scheme is a pathway to expedite organ offering and acceptance processes where there is a high risk of organ discard. It is triggered if the organ is deemed untransplantable, has accrued significant ischaemic time, or there are multiple centre declines because of concern regarding donor or organ quality. There is no detriment in 5-year outcomes of the kidneys that are subsequently transplanted [2].

Despite this, the paucity of deceased donor organs means that rationing of transplantation is inevitable. Who should be permitted to benefit from kidney transplantation? The metric most commonly used to evaluate a transplant programme is graft and patient survival, but are there reasonable alternative outcome measures? Might patients’ priorities diverge from those of the healthcare professionals? How valid is quality of life rather than quantity of life? We report a case that challenges current perspectives.

In 1985 a 15-year-old girl presented with end stage renal disease and commenced haemodialysis. After early graft failure of a first transplant, in 1987 she received a second deceased donor transplant which worked well. It failed after 36 years in 2023 (when the kidney was 95 years old). Haemodialysis recommenced via a left brachiocephalic arterio-venous fistula (AVF).

Her clinical course was significant for squamous cell carcinoma (SCC): 2013 local excision of vaginal intraepithelial neoplasia, 2019 radiotherapy for anal SCC followed in 2022 by rectal resection, 2021 right hemi-maxillectomy and radiotherapy for maxillary SCC. Furthermore, she had multiple non-melanoma skin lesions.

The patient was exceptionally keen for a third transplant. She struggled psychologically with dialysis-dependency, and its impact not only on her but also her two school-aged children and partner. Physiologically she was much older than her chronological 53 years, and a higher risk candidate from anaesthetic and surgical perspectives. A negative impact on recurrent malignancy of enhanced immunosuppression was considered inevitable.

More broadly there was ethical concern about organ utility, given her anticipated life expectancy. This apprehension, along with the modification of the risk-benefit ratio afforded by living donation, meant the team would only consider transplantation if there was an available well-matched living donor. However the patient was very highly sensitized and both volunteers were HLA incompatible. Listing for deceased donor transplantation was deferred.

In 2024 repeated intervention was required for her AVF, but in due course no further salvage was possible. Central venous stenoses meant emergency access was achieved via a femoral catheter.

Within 24 h there was an offer of both kidneys from a 76-year-old (donation after circulatory death) donor via the UK Fast Track scheme. There had been an out of hospital cardiac arrest with an hour downtime. Pre-mortal morbidities included hypertension, diabetes mellitus, and vascular disease. The kidneys were declined by all UK transplant centres.

Complexity was further increased by the presence of (i) a new left maxillary lesion for which diagnostic imaging and biopsy were awaited (ii) donor specific HLA antibodies at least historically (no up to date testing as patient not on the transplant waiting list).

If the current HLA profile was comparable to previously, it was anticipated the crossmatch would be currently negative, historically positive. There was not time for a physical crossmatch prior to transplant given the characteristics of this particular donor. A session of plasma exchange was possible while waiting for current HLA antibody testing results and for kidneys to arrive.

The questions considered and discussed openly with the patient included: will this (these) kidney(s) work sufficiently to provide dialysis-independence? Should it be single or dual transplant? Is the risk of rejection too great? Will transplantation shorten life? Will transplantation enhance life? What is the alternative in terms of vascular access?

The patient accepted that definitive answers could not be provided. On reflection, she was accepting of the risks and keen to proceed to transplantation.

A right renal kidney with ostial atherosclerosis of all three arteries and multiple cysts (the largest 7 cm lower pole) was received and transplanted. Histology of a suspicious cyst excised prior to implant, returned as benign. The kidney began to function after 3 days. No further dialysis was required. There was no rejection. The baseline serum creatinine achieved was ∼1.4 mg/dL (Figure 1).

**FIGURE 1:**
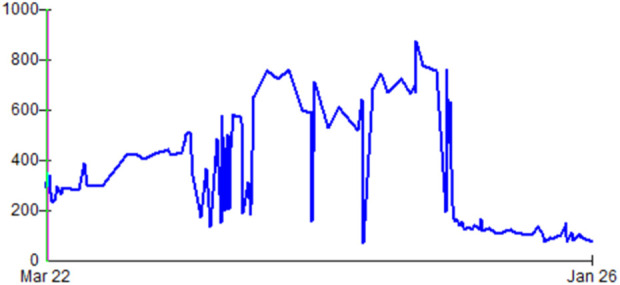
Trend in serum creatinine (umol/L) from failing second transplant, haemodialysis from March 2023, and subsequent transplant in December 2024.

The left maxillary lesion was biopsied at time of transplant, and demonstrated malignant squamous cells. Curative resection was not feasible, and treatment with radiotherapy and chemotherapy was initiated. Immunosuppression was tapered quickly and sirolimus substituted for tacrolimus and mycophenolate. There was no rejection. Hospitalisation was required twice for intravenous antibiotic therapy (during chemotherapy treatment). After some months there was escape of control of malignancy with progressive local and disseminated disease. She died at home 13 months after transplantation.

If palliative care is defined as “optimizing quality of life, and mitigating or reducing suffering among people with serious, complex, and often terminal illnesses” this was a palliative transplant. Quality of life was improved, and dialysis-associated suffering reduced, for this patient in her final year of life. Economically, transplantation was also advantageous compared to hospital-based haemodialysis [3] particularly as her course was uncomplicated and readmission was not required.

Did the transplant accelerate her death? And if so, was freedom from dialysis a reasonable exchange?

There was a substantial risk that death from vascular access failure would have happened more swiftly than death from malignancy. Early and aggressive tapering in anti-rejection medication was possible despite the intermediate immunological risk as there was no evidence of an immunological response. Within a few months the regimen was prednisolone and sirolimus only. Sirolimus was chosen given that it reduces the incidence of cutaneous SCC in renal transplant recipients [4], and it may have had additional benefit over and above minimisation of immunosuppression. Exogenous erythropoietin was no longer required (its pleiotropic effects may potentiate malignancy) and this could have been beneficial in survival [5]. Importantly, having reasonable kidney function meant that all chemotherapeutic options were available.

The patient was delighted with freedom from dialysis, this facilitated travel and holidays with her family in the final year of life. Having accepted a potentially poor outcome given the donor characteristics and immunological status, she was immensely grateful for what she considered an excellent transplant outcome.

Given that this kidney would otherwise have been discarded, nobody else was disadvantaged by transplanting it into someone with limited life expectancy. It raises important questions for the transplant community. Is quality of life a valid outcome measure in transplantation? Should marginal deceased donor organs, living donor kidneys, and xenotransplantation be considered for palliation? We welcome future debate about these important issues.

## Data Availability

The original contributions presented in the study are included in the article/supplementary material, further inquiries can be directed to the corresponding author.

## References

[1] NHS Blood and Transplant Annual (2025). Activity report 2024-25 activity-report-2024-2025-final.pdf page 34

[2] NHS Blood. NHS blood and transplant minutes of the fifty-first kidney advisory Group June 2025 UK TRANSPLANT (2025).

[3] Roberts G Holmes J Williams G Chess J Hartfiel N Charles JM Current costs of dialysis modalities: a comprehensive analysis within the United Kingdom. Perit Dial Int (2022) 42(6):578–84. 10.1177/08968608211061126 35068280

[4] Foerster Y Palaras J Mayer K Biedermann T Persa OD . Sirolimus for secondary prevention of cutaneous squamous cell carcinoma in kidney transplant recipients: a systematic review and meta-analysis of randomized controlled trials. Int J Dermatol (2026) 65:952–62. 10.1111/ijd.70285 41546605 PMC13067330

[5] Khuri FR . Weighing the hazards of erythropoiesis stimulation in patients with cancer. N Engl J Med (2007) 356(24):2445–8. 10.1056/NEJMp078101 17568023

